# Evaluation of Effectiveness of Wavelet Based Denoising Schemes Using ANN and SVM for Bearing Condition Classification

**DOI:** 10.1155/2012/582453

**Published:** 2012-11-14

**Authors:** Vijay G. S., Kumar H. S., Srinivasa Pai P., Sriram N. S., Raj B. K. N. Rao

**Affiliations:** ^1^Department of Mechanical and Manufacturing Engineering, Manipal Institute of Technology, Manipal University, Karnataka, Manipal 576104, India; ^2^Department of Mechanical Engineering, NMAM Institute of Technology, Karnataka, Nitte 574104, India; ^3^Department of Mechanical Engineering, Vidya Vikas Institute of Engineering and Technology, Karnataka, Mysore 570028, India; ^4^COMADEM International, Birmingham B29 6DA, UK

## Abstract

The wavelet based denoising has proven its ability to denoise the bearing vibration signals by improving the signal-to-noise ratio (SNR) and reducing the root-mean-square error (RMSE). In this paper seven wavelet based denoising schemes have been evaluated based on the performance of the Artificial Neural Network (ANN) and the Support Vector Machine (SVM), for the bearing condition classification. The work consists of two parts, the first part in which a synthetic signal simulating the defective bearing vibration signal with Gaussian noise was subjected to these denoising schemes. The best scheme based on the SNR and the RMSE was identified. In the second part, the vibration signals collected from a customized Rolling Element Bearing (REB) test rig for four bearing conditions were subjected to these denoising schemes. Several time and frequency domain features were extracted from the denoised signals, out of which a few sensitive features were selected using the Fisher's Criterion (FC). Extracted features were used to train and test the ANN and the SVM. The best denoising scheme identified, based on the classification performances of the ANN and the SVM, was found to be the same as the one obtained using the synthetic signal.

## 1. Introduction

The detection of fault in the machinery, in its incipient stage itself, has gained prime importance as it avoids machine down time, catastrophic failure of the machinery, threat to human life, high maintenance costs, and so forth. The fault diagnostic techniques based on the vibration signal analysis have become popular in recent times [1, 2]. The problem of the strong noise components masking the weak characteristic signals has always posed challenges to the condition monitoring expert. Several wavelet based signal processing techniques aiming at denoising the measured signal so as to increase the Signal-to-Noise Ratio (SNR) and reduce the Root-Mean-Square Error (RMSE) have been proposed and tried by several researchers [3–7]. The details of the techniques used by some of the researchers have been explained in Section 2.2. The wavelet based denoising technique has gained popularity due to its effectiveness and ease of application [8]. It overcomes the difficulty of determining the resonant frequency of the system. Therefore, the wavelet technique has been adopted in this work for denoising the bearing vibration signals. The detail coefficients, obtained from the Discrete Wavelet Transform (DWT), generally include a large proportion of the high-frequency noise components along with some of the characteristic information of the machine fault. Suitable compression or suppression of these components would remove the noise. The suppressed detail coefficients can then be used along with the original approximation coefficients in reconstructing the decomposed signal, by using the Inverse Wavelet Transform (IWT), which would now be fairly free of the noise [9, 10].

The Artificial Neural Networks (ANNs) and the Support Vector Machines (SVMs) have been used to a large extent in the fault diagnosis problems with high success rates. The bearing vibration signals are nonstationary signals and hence a nonlinear mapping from the input space to the output space is required, which is successfully fulfilled by the classifiers like the ANN and the SVM. Several researchers have applied the ANN and the SVM to the bearing fault identification problem. Wang et al. [12] have used the ANN, with difference values of the autoregressive coefficients as inputs, in a rotating machinery fault identification problem. Zarei [13] has proposed to improve the diagnostic abilities of an ANN applied to a four-condition bearing classification problem by using the time domain features alone as the ANN inputs. Kankar et al. [14] have applied the ANN and the SVM to a five-condition ball bearing defect classification problem and obtained high classification accuracies. The SVM is a soft computing tool which performs the tasks executed by an ANN, but with a different approach. The SVM positions a hyper plane between the two classes of data, thus separating the data belonging to the two classes. The ANN's approach is to minimize the error on the training data set which is known as the empirical risk minimization, whereas the SVM's approach is based on the structural risk minimization, in which the upper bound of the generalization error is minimized [15]. Yang et al. [16] have used the energy features extracted from a number of Intrinsic Mode Functions as input vectors to the SVM classifier to diagnose the REB condition. Sugumaran et al. [17] have illustrated the use of a decision tree to identify the best features, extracted from bearing vibration signal, which were given as inputs to the Proximal Support Vector Machine (PSVM) and the SVM. They reported that the PSVM performed better than the SVM. The popularity of the ANN and the SVM classifiers in the REB diagnostics has motivated the authors of this paper to use them in this work.

The objective of any classifier like the ANN or the SVM is to attain a good generalization ability, that is, to exhibit high accuracies on the training and the test data. This calls for the optimal design of the ANN/SVM architecture. One of the requirements of designing an optimal ANN/SVM architecture is to reduce the input dimensionality, that is, to select a few predominantly sensitive features as inputs. This is known as the Dimensionality Reduction Technique (DRT). Researchers have proposed and tried several DRTs. Some of the popular DRTs are Principal Component Analysis (PCA), Fisher's Criterion (FC), Singular Value Decomposition (SVD), Genetic Algorithm (GA), and so forth. Yen and Lin [20] have investigated the effectiveness of the DRTs, namely, Linear Discriminant Analysis and FC for reducing the number of wavelet packet features extracted for analyzing a bearing classification problem. Fuente et al. [21] have used the Fisher's Discriminant Analysis (FDA) for identifying the faults in a real plant in terms of maximizing the scatter between the classes and minimizing the scatter within each class. Chiang et al. [22] and Tang and Li [23] explain the fault diagnosis based on the FDA. Jack and Nandi [24], Samanta et al. [25], and Saxena and Saad [26] have shown in their works that the GA can be effectively used as a DRT along with the optimization of the topology parameters of the ANN/SVM. From the preliminary work carried out by the authors of this paper, it was found that the GA effectively selected the sensitive features, but took a longer time as the GA depended on the performance of the ANN or the SVM to compute the fitness value, that is, for every computation of the fitness value, the ANN or SVM had to be run, making the process time consuming. However, the effectiveness of the FC in selecting the sensitive features was found to be comparable with that of the GA based feature selection, and, more importantly, unlike the GA, FC was independent of the performance of the ANN or SVM. Therefore, in this work, FC has been used as a DRT.

**Figure 1:**
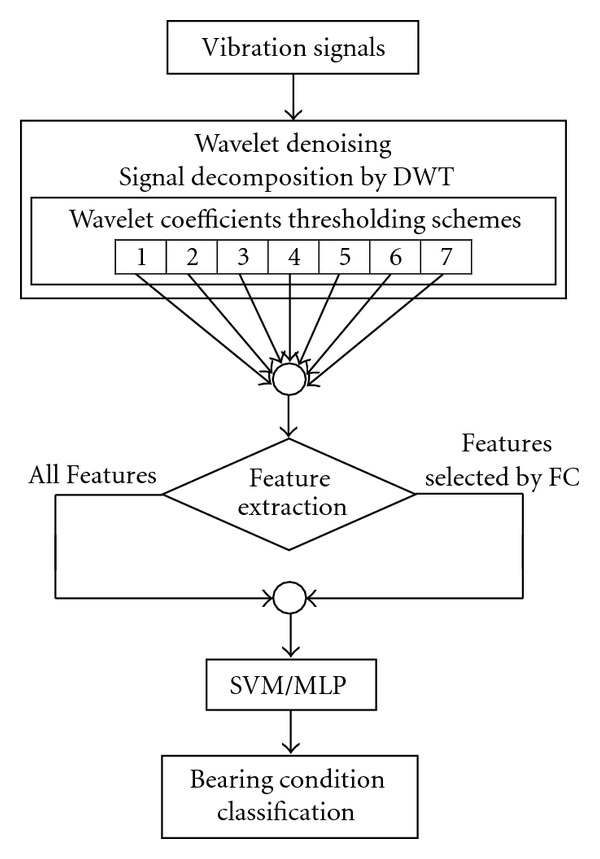
Denoising schemes and bearing diagnostic procedure employed in the study.

In this paper, the effectiveness of seven different wavelet based denoising schemes have been evaluated in terms of the classification accuracies of the ANN and the SVM on the denoised training and the test data, extracted from the REB vibration signals. Firstly, a synthetic signal (representing the vibration signal of a defective bearing) has been corrupted by a Gaussian white noise and subjected to the seven denoising schemes. Secondly, the real-time bearing vibration signals, measured from a customized bearing test rig under one load and two speed conditions, for four conditions of the bearings, have been subjected to the same denoising schemes. The denoising scheme which provided high SNR and low RMSE in the first part of the work provided high classification accuracies (on the training and the test data) in the second part of the work. The focus of this work was to evaluate the best wavelet based denoising scheme based on the performance of the ANN and the SVM.  Figure 1 shows the denoising schemes and the bearing diagnostic procedure employed in this study.

## 2. Wavelet Based Denoising 

The characteristic vibration signals of the defective bearings are not generally readily available when collected by means of a Data Acquisition (DAQ) system. This is mostly because the noise, influenced by the resonant frequency of the rotating system, masks the characteristic vibration signals. The noises are often stochastic signals whose frequency band will overlap with the interested signals. Therefore, it is difficult to eliminate the noise from the signals effectively by using the traditional filtering methods. Also, the traditional methods of denoising need the knowledge of the parameters which are difficult to be determined [8]. The wavelet based denoising has gained popularity due to its effectiveness and also that it overcomes the difficulties of the traditional denoising methods. The SNR must appreciably increase and the RMSE must become small on a successful application of a denoising method.

Suppose that a signal of interest *f*(*n*) has been corrupted by the noise *z*(*n*), so that we get a signal *g*(*n*) as in (1) which resembles the raw signal collected by means of a DAQ system,

(1) $$\begin{array}{c} g\left(n\right)=f\left(n\right)+\sigma z\left(n\right), \end{array}$$

where *z*(*n*) is a unit-variance, zero-mean Gaussian white noise and *σ* ^2^ is the variance of the noise. The denoising is a way to recover *f*(*n*) from the samples of *g*(*n*) as properly as possible. The three-step procedure adopted in the wavelet based denoising is (i) decomposition of the raw signal using the wavelet transform to get the approximation and the detail coefficients, (ii) suppressing the detail coefficients by selecting a suitable threshold value and by applying a suitable thresholding rule, and (iii) reconstructing the signal by applying IWT to the original approximation coefficients and the suppressed detail coefficients to get the denoised signal [9, 10].

Several denoising schemes (step ii) have been proposed by researchers [3–7]. In this work, the denoising effectiveness of seven different denoising schemes has been compared.  Table 1 gives the list of seven denoising schemes and the researchers who have proposed them.

### 2.1. Conventional Denoising Schemes

The wavelet denoising method focuses on the selection of the thresholding rules and the determination of the threshold value. Donoho [11] gave two thresholding rules, namely, hard-thresholding (*s*1) and the soft-thresholding (*s*2), which are considered to be the conventional wavelet based denoising schemes and they are readily available functions in the Wavelet toolbox of MATLAB. The hard-thresholding scheme is expressed as

(2) $$\begin{array}{c} y_{1}\left(x\right)=\{\begin{array}{cc} x & \text{if}\;\;\left|x\right|\geq \lambda ,\\ 0 & \text{if}\;\;\left|x\right|<\lambda , \end{array} \end{array}$$

where *x* is the wavelet coefficient, *y*_1_(*x*) is the corresponding suppressed wavelet coefficient by hard-thresholding, and *λ* is the threshold value.

The soft-thresholding scheme is expressed as

(3) $$\begin{array}{c} y_{2}\left(x\right)=\{\begin{array}{cc} \mathrm{sign}\left(x\right)\left(x-\lambda \right) & \text{if}\;\;\left|x\right|\geq \lambda ,\\ 0 & \text{if}\;\;\left|x\right|<\lambda , \end{array} \end{array}$$

where *y*_2_(*x*) is the suppressed wavelet coefficient obtained by soft-thresholding and the other terms have the same meaning as in (2).

### 2.2. Modified Soft-Thresholding Schemes Proposed by Different Researchers

A list of the denoising schemes *s*3 to *s*7 proposed by various researchers [3–7] is provided in Table 1. Huaigang et al. [3] have proposed an improved soft-thresholding function as given in (4). According to them, the conventional thresholding functions set the coefficients below the threshold value to zero, but, in their proposed method, these coefficients were tuned by a polynomial function. The coefficients that were below the threshold value and close to it were attenuated to a value less than the far coefficients. For important coefficients, the function was garrote-like, resulting in a more powerful function:

(4) $$\begin{array}{c} y_{4}\left(x\right)=\{\begin{array}{cc} x+\left(k-1\right)\lambda -0.5\frac{k\lambda^{m}}{x^{m-1}} & \text{if}\;\;x>\lambda ,\\ 0.5\frac{k{\left|x\right|}^{\left[m+\left(2-k\right)/k\right]}\;}{\lambda^{\left[m+\left(2-2k\right)/k\right]}}\mathrm{sign}\left(x\right) & \text{if}\;\;\left|x\right|<\lambda ,\\ x-\left(k-1\right)\lambda -0.5\frac{k\left(-\lambda^{m}\right)}{x^{m-1}} & \text{if}\;\;\left|x\right|<-\lambda , \end{array} \end{array}$$

where *m* and *k* are tuning parameters and the other terms in (4) have the same meaning as in (2). By tuning the parameter *k*, the thresholding function can be between the hard- and the soft-thresholding functions. By tuning the parameter *m*, the near-optimum thresholding function is adjusted to the optimum one by applying small changes. As per [3], optimization of the parameter *k* works similar to a global search and optimization of the parameter *m* works like a local search in finding the best thresholding function. The authors in [3] have selected *m* = 2 and 4 and *k* ∈ [0, 1]. In this work, *m* = 4 and *k* = 0.8 have been selected. 

Fang and Huang [4] have proposed a wavelet trimmed thresholding scheme as given in (5) which was an improved version of the hard- and the soft-thresholding schemes:

(5) $$\begin{array}{c} y_{5}\left(x\right)=\{\begin{array}{cc} x\left(\frac{\;{\left|x\right|}^{\alpha }-\lambda^{\alpha }}{{\left|x\right|}^{\alpha }}\right), & \text{if}\;\;\left|x\right|\geq \lambda ,\\ 0, & \text{if}\;\;\left|x\right|<\lambda , \end{array} \end{array}$$

where *α* is a parameter and the other terms in (5) have the same meaning as in (2). They suggested that with careful tuning of the parameter *α* for a particular signal, a best denoising effect could be achieved. When *α* = 1, it was equivalent to the soft-thresholding and when *α* → *∞*, it was equivalent to the hard-thresholding. Accordingly, in this work, *α* = 5 has been chosen.

Lin and Cai [5] proposed a new threshold function given in (6) which had the advantage of the nonnegative dead zone thresholding:

(6) $$\begin{array}{c} y_{6}\left(x\right)=\{\begin{array}{cc} \sqrt{1-\beta^{2}}\mathrm{sign}\left(x\right)\left|x-\lambda \right|+\beta x, & \text{if}\;\;\left|x\right|\geq \lambda ,\\ 0, & \text{if}\;\;\left|x\right|<\lambda , \end{array} \end{array}$$

(6a) $$\begin{array}{c} \beta =\exp\left[-\frac{{\left(\left|x\right|-\lambda \right)}^{2}}{k}\right], \end{array}$$

where *k* is a positive number and the other terms in (6) have the same meaning as in (2). When *x* → *λ*, *β* → 1 and *y*_6_(*x*) = *x*, which overcomes the disadvantage of the soft-thresholding, and when *x* → *∞*, *β* → 0 and *y*_6_(*x*) → 0, which makes the signal smoother than the hard-thresholding function. A value of *k* = 0.5 has been chosen in this work, for using this scheme.

Zhang et al. [6] have proposed an improved thresholding function given in (7):

(7) $$\begin{array}{c} y_{7}\left(x\right)=\{\begin{array}{cc} \mathrm{sign}\left(x\right){\left({\left|x\right|}^{u}-\alpha \lambda^{u}\right)}^{1/u}, & \text{if}\;\;\left|x\right|\geq \lambda ,\\ 0, & \text{if}\;\;\left|x\right|<\lambda , \end{array} \end{array}$$

where *u* and *α* are parameters whose proper tuning can provide an effective denoising while the other terms in (7) have the same meaning as in (2). The power *u* is used in order to enlarge the difference between the signal and the noise, *u* > 0 (*u* = 2,3, 4,…). It can be observed that when *α* = 0, (7) becomes the hard-thresholding function. When the value of *α* is appropriately chosen between 0 and 1, the effectiveness of the denoising could be optimized. For using this scheme in this work, *u* = 10 and *α* = 0.6 have been chosen.

 In a new thresholding function proposed by Cai-lian et al. [7], suppression of the detail coefficients was done according to (8):

(8) $$\begin{array}{c} y_{8}\left(x\right)=x\times \left\{\frac{1-\exp\left[-{\left(x/\lambda \right)}^{2}\right]}{1+\exp\left[-{\left(x/\lambda \right)}^{2}\right]}\right\}. \end{array}$$

This thresholding function depends on the proper selection of the constant *λ*. It is continuous unlike the conventional soft-thresholding function and is easily differentiable, statistically very reliable, and robust, making it completely suitable for the discrete signal denoising. The optimum value of *λ* can be determined as proposed in [7], but, in the current work, *λ* = 0.8 has been chosen by trial and error so as that the ANN's and the SVM's training and test accuracies were maximum.

## 3. Wavelet Based Denoising of a Synthetic Signal 

**Figure 2:**
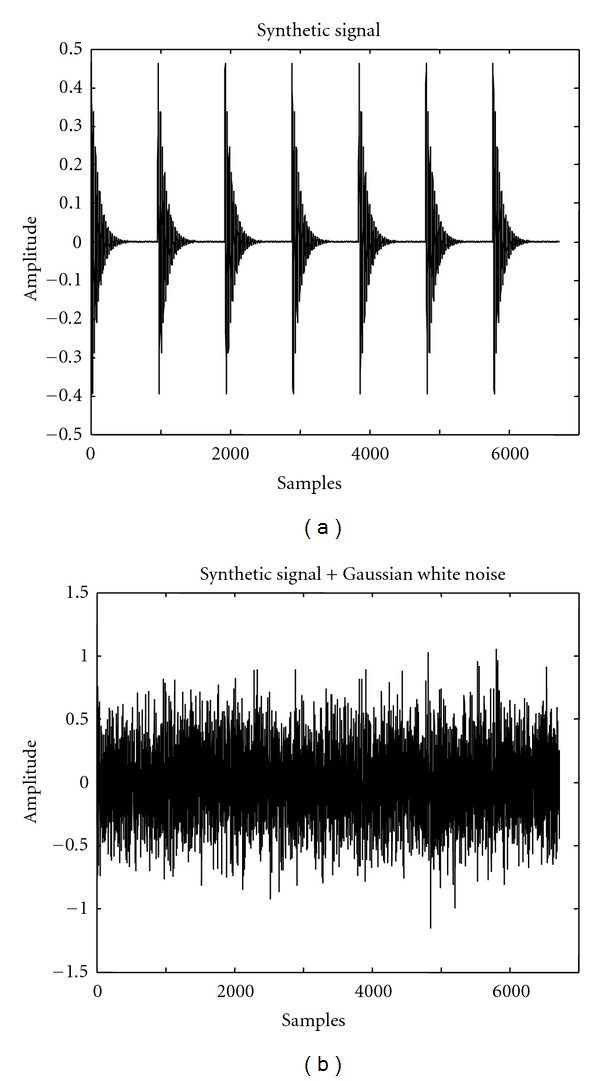
(a) Plot of a synthetic signal simulating a defective bearing vibration signal free of noise. (b) Plot of a signal corrupted with zero-mean Gaussian white noise.

The focus of the first part in this work was to apply the seven wavelet based denoising schemes listed in Table 1 to a synthetic signal that represented a defective bearing vibration signal. In order to simulate the vibration signal of a defective bearing, a weak synthetic signal 0.5*e* ^−500*t*^sin(10000*t*) with a defect frequency of 50 Hz was considered. A sampling frequency of 48 kHz was used as the real-time bearing vibration signals were acquired at the same rate. A plot of the synthetic signal is shown in Figure 2(a). It was corrupted with a strong zero-mean Gaussian white noise. A plot of the corrupted signal is shown in Figure 2(b). The energy of the synthetic signal was 41.89 and that of the corrupted signal was 510.77. The expression for computing the signal energy is given in (9), whereas the expressions for computing the SNR and the RMSE for a denoised signal are given in (11), and (12) respectively:

(9) $$\begin{array}{c} E=\sum_{i=1}^{N}x_{i}^{2}, \end{array}$$

where *E* is the energy of the signal *x* and *N* is the length of the signal:

(10) $$\begin{array}{c} \text{SNR}=10\;\text{lo}{\text{g}}_{e}\left[\frac{\sum_{i=1}^{N}x_{i}^{2}}{\sum_{i=1}^{N}{\left(d_{i}-x_{i}\right)}^{2}}\right], \end{array}$$

(11) $$\begin{array}{c} \text{RMSE}=\sqrt{\frac{1}{N}\sum_{i=1}^{N}{\left(d_{i}-x_{i}\right)}^{2}}, \end{array}$$

where *x* is the corrupted signal, *d* is the denoised signal, and *N* is the length of the signal.

**Figure 3:**
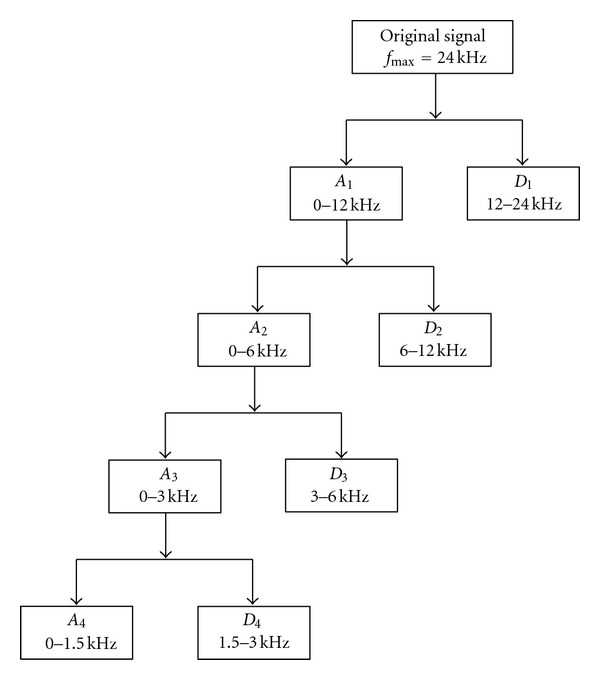
Discrete wavelet decomposition of bearing vibration signal.

**Figure 4:**
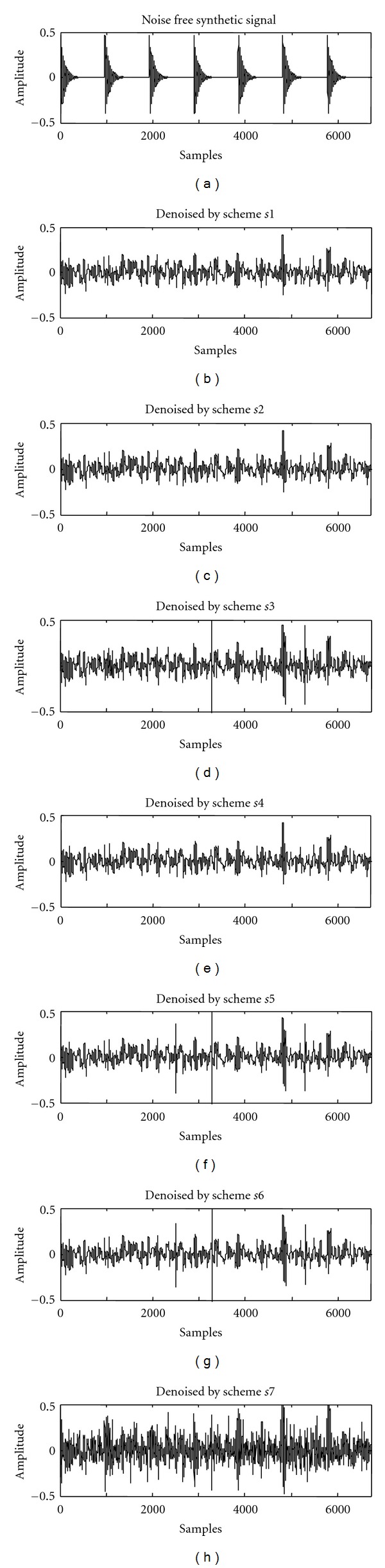
Plots of noise free synthetic signal and denoised signals by different schemes.

 The corrupted signal was subjected to the seven wavelet based denoising schemes listed in Table 1. The corrupted signal was subjected to the DWT so as to decompose it into four levels using Daubechies 8 mother wavelet through a customized MATLAB program. According to Nyquist's rule, the maximum frequency of the vibration signal was set to 24 kHz because the sampling frequency was 48 kHz. The frequency bandwidths of the approximation and the detail coefficients of the wavelet decompositions are shown in Figure 3. For each level of the wavelet decomposition, the threshold value *λ* was determined as per Stein's Unbiased Risk Estimate (SURE), as SURE threshold selection rules are more conservative as expressed in [9]. This threshold value was used for all denoising schemes, except for *s*7, where it was selected by trial and error.  Figure 4 shows the plots of the noise free synthetic signal and the denoised signals by different schemes. The values of E, SNR, and RMSE for the denoised signals are given in Table 2. The objective of signal processing was to increase the SNR and lower the RMSE of a corrupted signal. From Table 2, it can be observed that *s*7 gave high SNR and low RMSE. The peaks in the original synthetic signal (representing the bearing fault impulses) were identifiable more clearly in *s*7 denoising scheme when compared to the other schemes (refer to Figure 4).

## 4. Wavelet Based Denoising of Real-Time Vibration Signal

**Figure 5:**
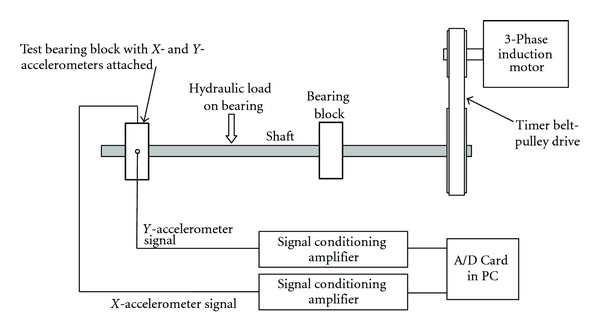
Schematic diagram of the test rig.

In the second part of the work in this paper, the objective was to apply the seven schemes of denoising discussed in the previous section to the vibration signals collected from a customized bearing test rig for four bearing conditions (*N*: normal bearing, IR: bearing with defect on inner race, *B*: bearing with defect on Ball, and OR: bearing with defect on an outer race). A schematic diagram of the customized test rig used for extracting the bearing vibration signals is shown in Figure 5. It consisted of a horizontal shaft mounted on a support bearing (right) and a test bearing (left). A radial load on the test bearing was applied through a hydraulic loading arrangement. The accelerometers were mounted on the horizontal and the vertical surfaces of the test bearing housing (*X* & *Y*). The vibration signals measured through the accelerometers were acquired at a rate of 48000 samples per second and stored in the computer through the DAQ system. The acceleration signals were collected for 5.08 seconds from a 6205 deep groove ball bearing under a radial load of 1.7 kN and shaft speeds of 356 and 622 rpm. The signals collected from accelerometer-*X* were considered for analysis, as the signals acquired in *Y*-direction were not very sensitive to the bearing condition. Each trial of the experiment resulted in a data vector of size 250000 × 1.

**Figure 6:**
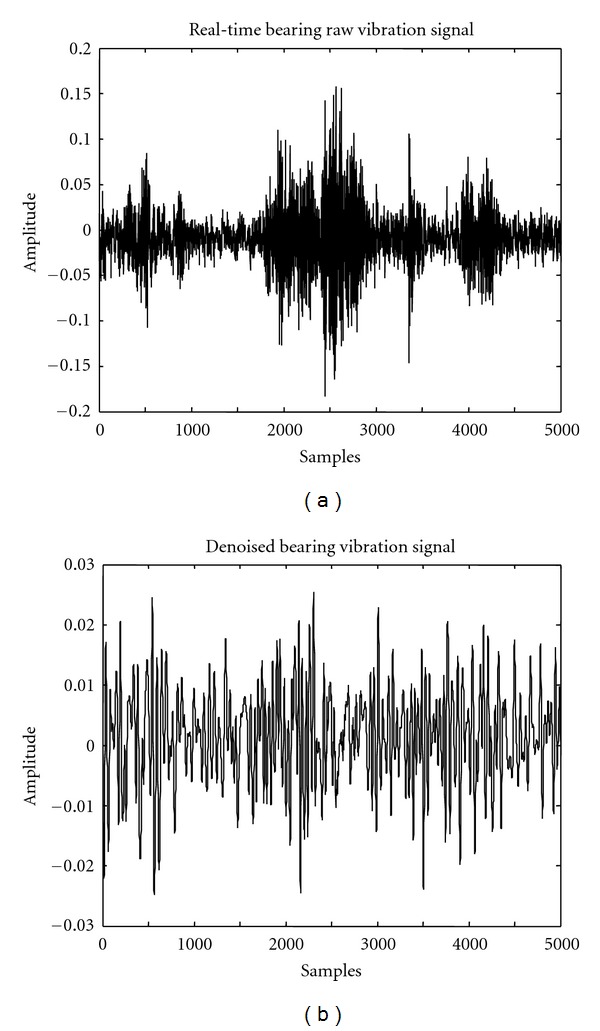
(a) Raw vibration signal from bearing with OR defect. (b) Denoised vibration signal from bearing with OR defect using *s*7.

Figure 6(a) shows the raw vibration signal collected from a bearing with the OR defect for a load of 1.7 kN and a speed of 622 rpm and Figure 6(b) shows the plot of the denoised signal using scheme *s*7. It is clear from the figures that the selected denoising scheme has been effective in representing the original signal with reduced noise (SNR-1.9279, RMSE 0.0261).

## 5. Feature Extraction

Each denoised vibration signal (250000 × 1) was divided into 50 nonoverlapping bins each with 5000 data. From each bin, 30 features were extracted out of which features 1 to 17 (*T*_1_ to *T*_17_) were the statistical time domain features and features 18 to 30 (*F*_1_ to *F*_13_) were the statistical frequency domain features. This formed a single pattern. Hence, for four conditions of the bearing, two speed conditions and one load condition, a total of 400 patterns (50 × 8) were extracted. The feature set matrix consisted of 30 features × 400 patterns. Each feature was normalized, by dividing each element of the feature by the feature maxima, so as to attain values between 0 and 1. The patterns of the matrix were thoroughly mixed, out of which 300 patterns (75%) were used in the training data set and the remaining 100 patterns (25%) in the test data set. A list of the features extracted from the denoised vibration signal is given in Table 3.

## 6. Feature Dimensionality Reduction

The FC has been used as a DRT in this work. The criterion for the FC is based on computing the “separation distance” between the two classes of interest and it depends upon the mean and the standard deviation of the two classes. The separation distance between the two classes as per the FC is given in (12) as suggested by Yen and Lin [20]:

(12) $$\begin{array}{c} J_{k}^{P,Q}=\frac{{\left|\text{Mean}\left(t_{k}^{P}\right)-\text{Mean}\left(t_{k}^{Q}\right)\right|}^{2}}{{\left[\text{Std}\left(t_{k}^{P}\right)\right]}^{2}+{\left[\text{Std}\left(t_{k}^{Q}\right)\right]}^{2}}, \end{array}$$

where *J*_*k*_^*P*,*Q*^ is a measure of the Fisher's Separation Distance between the two classes of the bearing *P* and *Q* for the *k*th feature (*P* and *Q* each may be *N*, IR, *B,* and OR defect). Mean() and Std() are the mean and the standard deviation. For the four-class problem in this study, the summation of the pairwise combinations *J*_*k*_^*N*,IR^, *J*_*k*_^*N*,OR^, *J*_*k*_^*N*,*B*^, *J*_*k*_^IR,OR^, *J*_*k*_^IR,*B*^, and *J*_*k*_^OR,*B*^ has been taken to estimate the Fisher's Discriminant Power (FDP) *F*_*k*_ of a specific feature *t*_*k*_ [20]:

(13) $$\begin{array}{c} F_{k}=J_{k}^{N,\text{IR}}+J_{k}^{N,\text{OR}}+J_{k}^{N,B}+J_{k}^{\text{IR},\text{OR}}+J_{k}^{\text{IR},B}+J_{k}^{\text{OR},B}, \end{array}$$

where *F* is a vector of FDPs.

 The features with higher values of the FDPs form sensitive inputs to the ANN/SVM classifiers. The FDPs computed for all the 30 features have been arranged in a descending order, resulting in a vector F*. In this paper a new method of selecting the number of sensitive features based on a threshold value **θ** has been proposed. The expression to compute **θ** is given in (14):

(14) $$\begin{array}{c} \theta \approx \frac{\sum_{k=1}^{s}F_{k}^{*}}{\sum_{k=1}^{f}F_{k}}, \end{array}$$

where *f* is the total number of features extracted (30 in this work) and *s* is the number of selected features such that the sum of the *s* largest FDPs divided by the sum of all the FDPs is approximately equal to **θ**. In this work, a threshold value of **θ** = 0.85 was chosen.  Table 4 shows the FDPs for signals denoised by seven schemes. It can be seen that different denoising schemes have selected different numbers of features (*s*) based on the threshold value of **θ** = 0.85.

## 7. Performance of the Denoising Schemes**  **Based on the ANN/SVM

In order to evaluate the performance of the different denoising schemes, the ANN/SVM classifiers were trained and tested using two types of inputs based on the features extracted from the denoised signals, namely, (i) the use of all the 30 features as inputs and (ii) the use of the features selected by the FC as inputs. A binary scheme of classification was used to define the bearing condition at the output of both the ANN and the SVM, namely, *N* (1 0 0 0), IR (0 1 0 0), *B* (0 0 1 0), and OR defect (0 0 0 1) to denote the four-class condition of the bearings.

### 7.1. ANN Classifier

**Figure 7:**
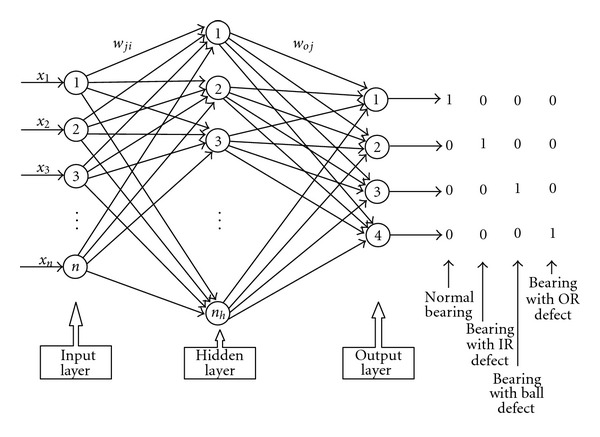
Structure of the MLPNN architecture.

The ANN adopted here was the Multilayer Perceptron Neural Network (MLPNN) which uses back propagation algorithm for training. Only one hidden layer with different numbers of neurons in hidden layer, *n*_*h*_ = 5, 10, 15, 20, 25 and 30 were used. The sigmoid activation function is used in the hidden and the output layer. A mean square error of 10^−6^, a minimum gradient of 10^−10^, and maximum number of epochs of 500 are used. The training process would stop if any one of these conditions were met. The initial weights and biases of the network were fixed randomly. The MLPNN was implemented by using the MATLAB Neural Network Toolbox.  Figure 7 shows the structure of the MLPNN used, where *x*_1_, *x*_2_,…*x*_*n*_ are the inputs (features), *n*_*h*_ are the number of nodes in the hidden layer, and *w*_*ji*_ and *w*_*oj*_ are the connection weights between the input-hidden layers and the hidden-output layers, respectively.

 The performance of the MLPNN classifier for the two types of inputs (all the features and the features selected by FC) extracted from the denoised signals is shown in Table 5. It is clear from the table that, for signals denoised using scheme *s*7, the accuracies on training and test data were higher compared to other schemes. The accuracies for different numbers of neurons in the hidden layer, *n*_*h*_ = 15, 20, 25, and 30 were comparatively lower and therefore have not been reported in the table.

### 7.2. SVM Classifier

The SVM classifier used in this work was based on the customized MATLAB tool box provided in [27]. In this work, the SVM was trained and tested for different values of the regularization parameter **γ** and the kernel width *σ* ^2^. Parameter **γ** was varied from 6 to 10 in steps of 1 and *σ* ^2^ was varied from 3 to 4 in steps of 0.25.

 The performance of the SVM classifier for the two types of inputs (all the features and the features selected by the FC) extracted from the denoised signals is shown in Table 6. It is clear from the table that, for signals denoised using scheme *s*7, the accuracies on the training and the test data were higher compared to the other schemes. The accuracies for the other values of **γ** and *σ* ^2^ were comparatively lower and are therefore not reported in the table.

## 8. Discussion

The focus of this work is to evaluate seven different wavelet based denoising schemes. In order to ascertain the effectiveness of the schemes, a corrupted synthetic signal simulating the real-time bearing vibration signal is used. By observation and also based on the SNR and RMSE, scheme *s*7 is found to be the most effective in denoising the corrupted signal. In order to evaluate its performance on a real-time bearing vibration signal, signals collected from bearings under four conditions and single load and two speeds were subjected to the same denoising schemes. The denoised signals were used for extracting the features in the time domain and the frequency domain. The features extracted were subjected to dimensionality reduction using the FC. Both the reduced feature set selected by the FC and the complete feature set were used as inputs to the ANN and the SVM classifiers for comparing the performance of the different denoising schemes.  Table 7 shows the performance of the ANN and the SVM classifier for the denoised vibration signal using the *s*7 scheme. It is clear from the table that the denoising scheme *s*7 resulted in more than 95% accuracy on the test data using the ANN and more than 80% accuracy on the test data using the SVM. Also for *s*7 scheme, the reduced feature set obtained using FC resulted in a higher performance in terms of the test data accuracy and the number of epochs when compared to the use of all the features. The proposed method of selecting the number of sensitive features from the vibration data obtained using different denoising schemes based on a threshold **θ** has been successful. Therefore, the DRT like the FC can be effectively used in improving the performance of the ANN and the SVM classifiers. Hence *s*7 scheme is found to be effective in denoising the real-time vibration signals, when compared to the other denoising schemes. 

## 9. Conclusions

This paper presents the evaluation of the effectiveness of the wavelet based denoising schemes using the ANN and the SVM classifiers applied to the bearing condition classification problem. Seven different denoising schemes selected from an extensive literature survey were used for denoising a synthetic corrupted signal resembling a bearing vibration signal. Based on the SNR and the RMSE, the best denoising scheme was selected. This scheme has been applied, along with the other schemes for denoising the real-time vibration signals collected from an REB test rig for four different bearing conditions, one load and two speeds. The features extracted from the denoised signal in the time domain and the frequency domain have been used as inputs to the ANN and the SVM classifiers. In order to reduce the dimension of the feature set, FC is used and the reduced feature set is also used as inputs to the ANN and the SVM. The proposed method of selecting the reduced number of features based on the threshold **θ** is found to be effective. It is found that the best denoising scheme selected based on the synthetic signal performed better (in terms of the classification accuracies and the number of epochs) with the feature set extracted from bearing vibration signals, when compared to the other denoising schemes. Hence, it can be concluded that *s*7 scheme can be effectively used to denoise the bearing vibration signals for an efficient classification of its condition.

## Figures and Tables

**Table 1:** List of thresholding schemes.

Thresholding scheme		Researcher/s
Conventional thresholding schemes	*s1 *	Donoho [11]
	*s2 *	Donoho [11]

Modified or improved thresholding schemes	*s3 *	Huaigang et al. [3]
	*s4 *	Fang and Huang [4]
	*s5 *	Lin and Cai [5]
	*s6 *	Zhang et al. [6]
	*s7 *	Cai-lian et al. [7]

**Table 2:** Energy, SNR, and RMSE of synthetic signal, corrupted signal, and denoised signals.

Signal type	Energy	SNR	RMSE
Synthetic signal	41.895919	—	—
Corrupted signal	510.773031	0.793049	0.264839
Signal denoised by *s1 *	40.005523	0.824919	0.264418
Signal denoised by *s2 *	40.005523	0.824919	0.264418
Signal denoised by *s3 *	48.305217	1.539327	0.255139
Signal denoised by *s4 *	40.005523	0.824919	0.264418
Signal denoised by *s5 *	44.967623	0.930983	0.263019
Signal denoised by *s6 *	44.136735	0.930165	0.263030
Signal denoised by *s7 *	75.604166	4.712617	0.217706

**Table 3:** Time and frequency domain features extracted for the study.

Sl. number	Feature
(1)	$T_{1}=n^{-1}\sum_{i=1}^{n}x_{i}$
(2)	$T_{2}={\left[n^{-1}\sum_{i=1}^{n}x_{i}^{2}\right]}^{1/2}$
(3)	$T_{3}={\left[{\left(n-1\right)}^{-1}\sum_{i=1}^{n}{\left(x_{i}-T_{1}\right)}^{2}\right]}^{1/2}$
(4)	$T_{4}=\frac{1}{2}\left[\mathrm{Max}\left(x_{i}\right)-\mathrm{Min}\left(x_{i}\right)\right]$
(5)	$T_{5}={\left(n^{-1}\sum_{i=1}^{n}\sqrt{\left|x_{i}\right|}\right)}^{2}$
(6)	$T_{6}=n^{-1}\sum_{i=1}^{n}\left|x_{i}\right|$
(7)	$T_{7}={\left(n-1\right)}^{-1}\sum_{i=1}^{n}{\left(\frac{x_{i}-T_{1}}{T_{3}}\right)}^{3}$
(8)	$T_{8}={\left(n-1\right)}^{-1}\sum_{i=1}^{n}{\left(\frac{x_{i}-T_{1}}{T_{3}}\right)}^{4}$
(9)	$T_{9}=\frac{T_{4}}{T_{2}}$
(10)	$T_{10}=\frac{T_{4}}{T_{5}}$
(11)	$T_{11}=\frac{T_{2}}{T_{6}}$
(12)	$T_{12}=\frac{T_{4}}{T_{6}}$
(13)	$T_{13}=\frac{T_{3}}{T_{6}}$
(14)	$T_{14}={\left[\log\left(T_{3}\right)\right]}^{-1}\sum_{i=1}^{n}\log\left(\left|x_{i}\right|+1\right)$
(15)	$T_{15}=\sqrt{\frac{T_{5}}{T_{3}}}$
(16)	$T_{16}=-\sum_{i=1}^{n}\log\left\{{\left[T_{3}\sqrt{2\pi }\right]}^{-1}\exp\left[\frac{-{\left(x_{i}-T_{1}\right)}^{2}}{2T_{3}^{2}}\right]\right\}$
(17)	$T_{17}=-\sum_{i=1}^{n}\log\left[\beta \eta^{-\beta }{\left|x_{i}\right|}^{\beta -1}\exp{\left\{\frac{\left|x_{i}\right|}{\eta }\right\}}^{\beta }\right]$,
	where *β* is the shape factor and *η* is the scale factor
(18)	$F_{1}=N^{-1}\sum_{k=1}^{N}S\left(k\right)$
(19)	$F_{2}={\left(N-1\right)}^{-1}\sum_{k=1}^{N}{\left[S\left(k\right)-F_{1}\right]}^{2}$
(20)	$F_{3}=N^{-1}F_{2}^{-1.5}\sum_{k=1}^{N}{\left[S\left(k\right)-F_{1}\right]}^{3}$
	
(21)	$F_{4}=N^{-1}F_{2}^{-2}\sum_{k=1}^{N}{\left[S\left(k\right)-F_{1}\right]}^{4}$
(22)	$F_{5}=\left[\sum_{k=1}^{N}f_{k}S\left(k\right)\right]{\left[\sum_{k=1}^{N}S\left(k\right)\right]}^{-1}$
(23)	$F_{6}={\left\{N^{-1}\sum_{k=1}^{N}{\left[f_{k}-F_{5}\right]}^{2}S\left(k\right)\right\}}^{1/2}$
(24)	$F_{7}={\left\{\left[\sum_{k=1}^{N}f_{k}^{2}S\left(k\right)\right]{\left[\sum_{k=1}^{N}S\left(k\right)\right]}^{-1}\right\}}^{1/2}$
(25)	$F_{8}={\left\{\left[\sum_{k=1}^{N}f_{k}^{4}S\left(k\right)\right]{\left[\sum_{k=1}^{N}f_{k}^{2}S\left(k\right)\right]}^{-1}\right\}}^{1/2}$
(26)	$F_{9}=\left[\sum_{k=1}^{N}f_{k}^{2}S\left(k\right)\right]{\left\{\sum_{k=1}^{N}S\left(k\right)\sum_{k=1}^{N}f_{k}^{4}S\left(k\right)\right\}}^{-1/2}$
(27)	$F_{10}=\frac{F_{6}}{F_{5}}$
(28)	$F_{11}=N^{-1}F_{6}^{-3}\sum_{k=1}^{N}{\left(f_{k}-F_{5}\right)}^{3}S\left(k\right)$
(29)	$F_{12}=N^{-1}F_{6}^{-4}\sum_{k=1}^{N}{\left(f_{k}-F_{5}\right)}^{4}S\left(k\right)$
(30)	$F_{13}=N^{-1}F_{6}^{-0.5}\sum_{k=1}^{N}{\left(f_{k}-F_{5}\right)}^{1/2}S\left(k\right)$
	

*Features 1 to 17 (*T*_1_ to *T*_17_) are statistical features extracted from data in time domain and 18 to 30 (*F*_1_ to *F*_13_) are features extracted from data in the frequency domain. *n* is the number of data points in the time domain signal, *x*_*i*_ is the acceleration amplitude of *i*th data point in the time domain signal, *N* is the number of lines in the frequency spectrum, *S*(*k*) is the amplitude of the *k*th line in the frequency spectrum, *f*_*k*_ is the frequency value of the *k*th line in the frequency spectrum [18, 19].

**Table 4:** FDP and selected features by FC.

FDPs arranged in a descending order for signals denoised by seven schemes													
*s*1		*s*2		*s*3		*s*4		*s*5		*s*6		*s*7	
*s* = 5		*s* = 5		*s* = 6		*s* = 6		*s* = 4		*s* = 4		*s* = 9	
#	F*	#	F*	#	F*	#	F*	#	F*	#	F*	#	F*
1	419.43	1	419.43	1	419.43	1	419.43	1	419.43	1	419.43	1	419.43
17	11.13	17	11.13	17	11.17	17	11.13	17	11.12	17	11.12	17	11.12
21	7.36	21	7.36	21	7.38	21	7.36	21	7.36	21	7.37	26	8.56
5	7.21	5	7.21	5	7.25	5	7.21	5	7.22	5	7.22	24	8.15
20	7.11	20	7.11	20	7.21	20	7.11					21	7.31
				6	6.33	6	6.31					5	7.16
												20	7.04
												22	6.94
												6	6.29

$\sum_{k=1}^{k=s}F_{k}^{*}$	452.24		452.24		458.77		458.55		445.13		445.14		482

$\sum_{k=1}^{k=f}F_{k}$	533.87		533.87		531.85		533.87		519.46		520.73		565.51

*θ*	0.85		0.85		0.86		0.86		0.86		0.85		0.85

**Table 5:** Performance of the MLPNN classifier.

Scheme	Number of neurons in hidden layer	All the 30 features as input			Features selected by FC as inputs		
		Epochs	Training accuracy (%)	Test accuracy (%)	Epochs	Training accuracy (%)	Test accuracy (%)
*s1 *	5	30	100	95.67	82	100.00	88.75
	10	35	100	92.67	78	100.00	89.75

*s2 *	5	36	100	93.67	200	98.83	92.25
	10	47	100	94.67	63	100.00	89.25

*s3 *	5	41	100	94.33	38	100.00	95.25
	10	61	100	90.33	163	100.00	90.50

*s4 *	5	15	100	96.00	153	99.92	91.75
	10	42	100	94.00	102	100.00	86.50

*s5 *	5	61	100	93.67	157	99.92	92.50
	10	182	100	86.00	92	100.00	88.00

*s6 *	5	207	99.11	90.00	152	99.92	92.25
	10	80	100	87.33	143	100.00	89.25

*s7 *	**5**	**22**	**100**	**97.67**	**18**	**100.00**	**95.50**
	**10**	**52**	**100**	**92.33**	**124**	**100.00**	**91.25**

**Table 6:** Performance of SVM classifier.

Scheme	All the 30 features as input				Features selected by FC as inputs			
	*γ*	*σ* ^2^	Training accuracy (%)	Test accuracy (%)	*γ*	*σ* ^2^	Training accuracy (%)	Test accuracy (%)
*s1 *	6	3.5	68.67	58.00	6	3	68.67	57.00
*s2 *	6	3.5	68.67	58.00	6	3	68.67	57.00
*s3 *	6	3.75	78.33	67.00	6	3	75.00	63.00
*s4 *	6	3.5	68.67	58.00	6	3	68.67	57.00
*s5 *	6	3.0	67.67	57.00	9	3	71.67	64.00
*s6 *	6	3.0	67.67	57.00	10	3	71.67	64.00
*s7 *	**7**	**3.5**	**86.00**	**80.00**	**8**	**3**	**85.33**	**84.00**

**Table 7:** The effectiveness of the denoising scheme *s7* using the ANN and the SVM.

	All feature set		Reduced feature set	
	Training accuracy (%)	Test accuracy (%)	Training accuracy (%)	Test accuracy (%)
ANN	100.00	97.67	100.00	95.50
SVM	86.00	80.00	85.33	84.00
